# Induction of high-titer IgG antibodies against multiple leukemia-associated antigens in CML patients with clinical responses to K562/GVAX immunotherapy

**DOI:** 10.1038/bcj.2013.44

**Published:** 2013-09-06

**Authors:** L Qin, B D Smith, H-L Tsai, N K Yaghi, P H Neela, M Moake, J Fu, YL Kasamon, G T Prince, M Goswami, G L Rosner, H I Levitsky, C S Hourigan

**Affiliations:** 1 Sidney Kimmel Comprehensive Cancer Center at Johns Hopkins, Baltimore, MD, USA; 2 Myeloid Malignancies Section, Hematology Branch, National Heart, Lung and Blood Institute, Bethesda, MD, USA; 3 Cancer Immunology Experimental Medicine, Pharma Research and Early Development, Roche Glycart AG, Schlieren, Switzerland

**Keywords:** leukemia, chronic myeloid, cancer vaccines, immunotherapy, K562 cells

## Abstract

The ability to target myeloid leukemia with immunotherapy would represent a significant therapeutic advance. We report here immunological analysis of clinical trials of primary and secondary vaccination with K562/GM-CSF immunotherapy in adult chronic phase chronic myeloid leukemia patients (CML-CP) with suboptimal responses to imatinib mesylate. Using serological analysis of recombinant cDNA expression libraries of K562 with autologous vaccinated patient serum, we have identified 12 novel chronic myeloid leukemia-associated antigens (LAAs). We show that clinical responses following K562/GM-CSF vaccination are associated with induction of high-titer antibody responses to multiple LAAs. We observe markedly discordant patterns of baseline and induced antibody responses in these identically vaccinated patients. No single antigen was recognized in all responses to vaccination. We demonstrate that an additional ‘booster' vaccination series can be given safely to those with inadequate responses to initial vaccination, and is associated with more frequent induction of IgG responses to antigens overexpressed in K562 vaccine compared with primary CML-CP. Finally, those with induced immune responses to the same LAAs often shared HLA subtypes and patients with clinical responses following vaccination recognized a partially shared but non-identical spectrum of antigens; both findings have potentially significant implications for cancer vaccine immunotherapy.

## Introduction

Cancer immunotherapy, in the form of allogeneic stem cell transplantation, has been used successfully to treat hematological malignancies for >40 years.^1^ The significant toxicity and logistical challenges associated with this treatment has however motivated the search for safer and more practical immunotherapy modalities (reviewed by Hourigan and Levitsky^2^). Targeted cellular therapy against cancer has the potential to be highly effective but requires either the ability to perform *ex vivo* augmentation of endogenous antitumor responses^3^ or prior knowledge of optimal cancer antigen targets.^4, 5^ These limitations of targeted adoptive cellular therapy, along with other factors, make vaccination^6^ and/or immune checkpoint inhibition^7, 8, 9^ potentially attractive approaches for cancer immunotherapy; unfortunately, these therapies are currently curative in only a minority of treated patients. Our understanding of the immunological underpinnings of those few observed clinical responses are still in their infancy.^10^

CML has been transformed over the past decade by the introduction of rationally designed tyrosine kinase inhibitors (TKIs) to an almost chronic disease with an estimated median survival from diagnosis of >20 years.^11^ However, significant clinical challenges remain, including difficulties with treatment adherence, side effects, contraindication in pregnancy, development of resistance mutations and the significant health-care costs of providing potentially lifelong therapy in a disease where the prevalence in the United States is predicted to increase six-fold from pre-TKI levels by 2035.^12^ Prior to the advent of TKIs, the best available treatments for CML involved immunotherapy,^13^ either interferon^14^ or stem cell transplantation.^15^ CML is known to be particularly susceptible to the immune control.^16, 17, 18, 19^ Detailed information regarding the specifics of this immune response to CML has come from the serologic screening of cDNA expression libraries (SEREX)^20^ in CML patients,^21, 22^ including patients with relapsed CML after SCT who achieved a complete remission after treatment with donor lymphocyte infusion.^23^ Finally, CML is a clonal stem cell disorder with a characteristic chromosomal translocation allowing highly sensitive detection of minimal residual disease.^24^ All these factors make CML an excellent model to investigate the mechanisms underlying clinical responses to immunotherapy in the hematological malignancies.

Peptide vaccination approaches in cancer have been repeatedly shown to be safe and capable of inducing anticancer antigen immune responses;^2, 25, 26^ however, questions remain regarding clinical efficacy and the practicality of leukemia clone-specific antigen identification and patient HLA-specific epitope validation.^27, 28^ In an attempt to overcome these limitations a variety of whole-cell vaccination approaches have been investigated, including the use of the irradiated allogeneic cancer cell lines genetically modified to express high levels of the immunomodulatory cytokine GM-CSF (‘GVAX').^29, 30, 31^ Clinical trials have been conducted using this vaccine platform in acute myeloid leukemia^32^ and pancreatic,^33^ breast,^34^ prostate,^35^ renal cell^36^ and non-small cell lung cancers.^37^ We have previously reported clinical responses following K562/GVAX vaccination in 19 patients with CML-CP with suboptimal responses to TKI therapy.^38^ In this study we report the development of a novel variation of the SEREX methodology (Quan-SEREX) that we use to identify induced IgG responses to leukemia-associated antigens (LAAs) associated with clinical responses following vaccination with K562/GVAX and to quantify changes in serum antibody titers of leukemia antigen-specific immunity over time.

## Materials and methods

### Clinical trials

J0345, ‘K562/GM-CSF Vaccination in Combination with Imatinib Mesylate for Chronic Myeloid Leukemia,' was a single-institution, pilot study performed at the Sidney Kimmel Comprehensive Cancer Center at Johns Hopkins (SKCCC).^38^ It was IRB-approved on 04 December 2003 and patients were accrued from January 2004 to August 2005. A total of 19 subjects were enrolled who had achieved at least a major cytogenetic response (<35% Ph+ hematopoiesis) but remained with evidence of measurable disease after at least 12 months of imatinib therapy. Prior to vaccination all subjects were followed up for 12 weeks to confirm the stability and persistence of either cytogenetic evidence of Philadelphia chromosome (Ph+) or molecular findings of measurable BCR/ABL fusion transcript by real-time quantitative PCR (RQ-PCR).

A subsequent trial, J0625, ‘K562/GM-CSF Vaccination in Combination with Imatinib Mesylate as Booster for Chronic Myeloid Leukemia Patients Previously Vaccinated on Protocol J0345,' was IRB-approved on 30 May 2006. Accrual at SKCCC began August 2006 and ended with the enrollment of the eleventh patient in December 2008. Eligible subjects were defined as those who had received all four vaccinations on J0345, completed follow-up observation period through week 36, had no dose-limiting toxicities or did not experience toxicity defined as severe adverse event, and had measurable residual CML after J0345. Disease burden was measured within 4 weeks of the planned vaccination boost.

**Figure 1:**
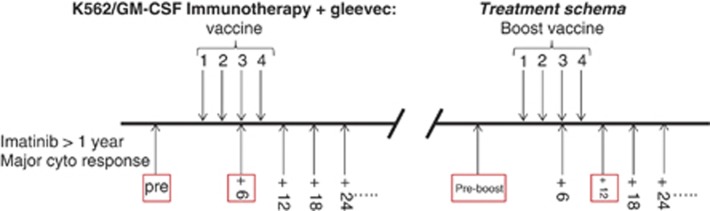
Clinical trials schema. We report here correlative immunological investigations of two consecutive immunotherapy pilot studies conducted at Johns Hopkins with patient accrual between 2005 and 2008. Left: the first trial (J0345) enrolled 19 patients with suboptimal clinical response after at least 1 year of imatinib for a study consisting of a 12-week pre-enrollment monitoring period, four vaccinations of irradiated K562/GM-CSF whole-cell vaccine given over a 9-week period followed by extended monitoring. Right: the second trial (J0625) offered a subset of patients with suboptimal clinical responses after the first vaccination series (*n*=11) a second series of 'booster' vaccinations given in the same manner. The mean time between the primary and booster vaccination series was 111 weeks (range 77–199 weeks). In both trials, vaccine product consisted of 1 × 10^8^ irradiated K562/GM-CSF cells (1.0 × 10^7^ cells per injection in 0.5 cc × 10 intradermal sites on the limbs) known to stably express >1000 ng of GM-CSF/10^6^ cells/24 h. Each injection was followed 3 hours later by the application of an adjuvant (toll-like receptor-7 agonist) 5% imiquimod cream (Aldara, Graceway Pharmaceuticals) on 9 of the 10 vaccination sites. All patients remained on imatinib therapy throughout both studies. The boxed time points were key for response assessment in this study.

On both of these trials, patients received a series of intradermal vaccinations each consisting of 1 × 10^8^ irradiated K562/GM-CSF cells, given every 3 weeks, for a total of four vaccinations. Imiquimod (Aldara, Graceway Pharmaceuticals, Bristol, TN, USA) was applied after vaccination to 9 of 10 injection sites. Imatinib was continued throughout the study period. Disease burden was measured every 3 months throughout the course of planned vaccination for 48 weeks following vaccination. Blood for immune assays was collected at 6-, 12-, 24-, 36- and 48-week visits (Figure 1).

### Serum samples

Blood samples from CML-CP patients and healthy donors were centrifuged at 2500 r.p.m. at 4 °C for 10 min and supernatants were stored at −80 °C. Patient sera were collected and cryopreserved at the same time points as the samples obtained for tumor burden assessment.

### Human K562 cell cDNA libraries

K562 cDNA phage library was constructed as described.^39^ Briefly, total RNA was prepared from K562 cells using Qiagen RNeasy Maxi column (Qiagen, Valencia, CA, USA). Poly (A)+ RNA was purified from total RNA with Invitrogen Micro-FastTrack mRNA isolation kit (Invitrogen, Carlsbad, CA, USA). Five micrograms of Poly (A)+RNA were used to synthesize cDNA. The cDNA library was constructed in a ZAP express vector and packaged into phage particles using ZAP-cDNA Synthesis Kit (Stratagene, Santa Clara, CA, USA) according to the manufacturer's instructions.

### K562 cDNA library screening

Serum samples from five patients with unambiguous clinical responses to initial GVAX/K562 vaccination were used to screen the cDNA expression library. Primary screening of the library was performed as previously described,^23, 39^ with minor modification. Briefly, 5 × 10^4^ recombinant phages per 15 cm dish were cultured at 42 °C for 4 h. Expression of recombinant protein was induced by incubation with isopropyl-D-thiogalactoside (IPTG)-treated nitrocellulose membranes for 8 h at 37 °C. After washing and blocking with 5% nonfat dry milk in TBST (100 mM Tris-HCl (pH 7.5), 0.15 M NaCl and 0.005% Tween 20), membranes were incubated overnight with phage/bacterial-depleted, week 12 post vaccine (1:200 dilution) patient sera followed by incubation with alkaline-phosphatase-conjugated goat anti-human IgG antibody (Jackson ImmunoResearch Laboratories, West Grove, PA, USA) diluted at 1:750 in TBST. Antigen–antibody complexes were visualized by staining with 5-bromo-4-chloro-3-indolylphosphatase and nitroblue tetrazolium (BCIP/NBT) (Invitrogen). Individual clones identified in membranes were plated for secondary and tertiary screenings using the same serum to obtain monoclone and retest for serum reactivity. The cDNA inserts of selected clones were excised and sequenced using T3 and T7 primers.

### Pre-post vaccine screen

Purified positive clones were mixed with nonreactive phage clone (β-gal phage) as an internal negative control at a 1:100 ratio. Two hundred microliters of XL1-blue *E. coli* bacteria was transfected with this mix and was plated in NZY agar plates for 4 h at 42 °C. Recombinant protein expression was induced by incubation with IPTG-treated nitrocellulose membranes for an additional 8 h at 37 °C. After washing in TBST and blocking overnight with 5% nonfat dry milk in TBS, each membrane was cut in half and incubated overnight with patient serum at 1:200 dilution from either pre-vaccination or week 12 post-vaccine time points, followed by incubation with alkaline-phosphatase-conjugated goat anti-human IgG antibody diluted at 1:750 in TBST. Antigen–antibody complexes were visualized by staining with BCIP/NBT. The intensity of positive staining was visually graded on a scale of 1–3. Changes of intensity were assessed between pre and post-vaccine serum.

### Quantitative immunoscreening assay (Quan-SEREX)

Fourteen vaccine-induced antigens identified from the pre-post immune screen were examined for their immunoreactivity and antigen-specific antibody titer in sera from 19 healthy donor and 19 CML patients (including five from the initial screening described above) at seven time points during the vaccine trial. Phage lysate of each of the 14 candidate antigens as well as β-gal and human IgG control antigen was diluted in SM buffer at 2 × 10^6^ pfu/μl and dotted in triplicates on XL1 bacterial lawn in 96-well format using 96-pin replicator (Molecular Devices, Sunnyvale, CA, USA), and incubated with IPTG-treated nitrocellulose membranes for 12 h at 37 °C. After washing in PBS and blocking overnight with 3% BSA in PBS, each membrane was incubated with individual serum at 1:200 overnight. The membranes were washed with PBS with 0.1% Tween 20 and incubated with infrared-labeled anti-human IgG at 1:10 000 dilution for 1 h at room temperature. Membranes were then washed three times in PBS/0.1% Tween 20 for 30 min each and then in PBS for 5 min. Dried membranes were scanned in Odyssey Infrared Imaging scanner (LI-COR, Lincoln, NE, USA).

### Statistical analysis

Observed intensities from Quan-SEREX immunoscreening of patient serum to any individual phage were normalized using the formula: (*V*x−*V*neg)/(*V*pos−*V*neg), where *V*x is the original raw intensity, *V*neg is the average intensity of the negative controls and *V*pos is the average intensity of the positive controls.

The association between recognition phages and clinical response to K562/GM-CSF immunotherapy was explored by examining the frequency of specific recognition phages among the 19 CML patients, as well as trend patterns between normalized intensities for individual recognized phages and BCR–ABL measurements over time. We performed statistical analyses in the R statistical environment (version 2.15.1).

### Quantitative real-time PCR

RNA was isolated from the K562 cell line and ficoll-purified peripheral blood mononuclear cells of eight CML patients (four chronic phase and four accelerated phase) and eight healthy donors using AllPrep DNA/RNA mini kit (Qiagen). RNA was assessed using a Nanodrop 1000 Spectrophotometer (Wilmington, DE, USA) and Agilent RNA 6000 Nano Kit and 2100 Bioanalyzer (Santa Clara, CA, USA). Subsequently, 400 ng of total RNA was reverse-transcribed using RT2 First Strand Kit (Qiagen). Custom RT^2^ Profiler PCR array plates (SABiosciences, Qiagen, Valencia, CA, USA) were used for PCR reactions performed using RT^2^ SYBR Green ROX qPCR Mastermix (SABiosciences) according to the manufacturer's instructions on an ABI 7900 thermal cycler (Applied Biosystems, Foster City, CA, USA), with a program of 10 min at 95 °C, followed by 40 cycles at 95 °C for 15 s and 60 °C for 1 min. Fold change expression values were calculated according to the comparative C(T) method^40^ using the geometric mean of three endogenous control genes (HPRT1, PPIH and TFRC).

## Results

### Patient characteristics

We reported previously the characteristics and the clinical outcomes for 19 subjects enrolled on an initial study (J0345) of K562/GVAX vaccine immunotherapy for CML-CP patients with persistent measurable disease despite at least 1 year of imatinib therapy.^38^ A subsequent clinical protocol (J0625) enrolled 11 of these subjects with suboptimal responses to the original vaccine series for a second ‘booster' series of K562/GVAX vaccinations. An overall schema of the two trials, including the time points used in this study for assessment of leukemia-specific immunity, is shown in Figure 1.

Offering a series of ‘booster' vaccinations to those patients who, after the initial vaccination series, did not have a molecular response or who had an initial molecular remission followed by return of measurable disease is based on the assumption that these patients do not have sufficient anti-leukemic immune response to cause eradication of their disease. Median age of patients on the J0625 ‘booster' study was 60. The average time between the last vaccination on J0345 and the initial vaccination on protocol J0625 was 111 weeks. The average time on imatinib prior to initiating booster vaccinations was 279 weeks. Patients getting booster vaccinations experienced similar local site reactions as they did when receiving the primary set of vaccines on the pilot study. No grade 3 or 4 adverse events were noted. All patients remained medically stable and tolerated the booster vaccinations well. Importantly, 8 of the 11 achieved their lowest recorded BCR–ABL RQ-PCR levels following the booster vaccination series; the median time to this value was 24 weeks. Two patients became completely molecularly negative for the BCR–ABL transcript following booster vaccination series (Table 1).

### SEREX for leukemia antigen discovery

**Figure 2:**
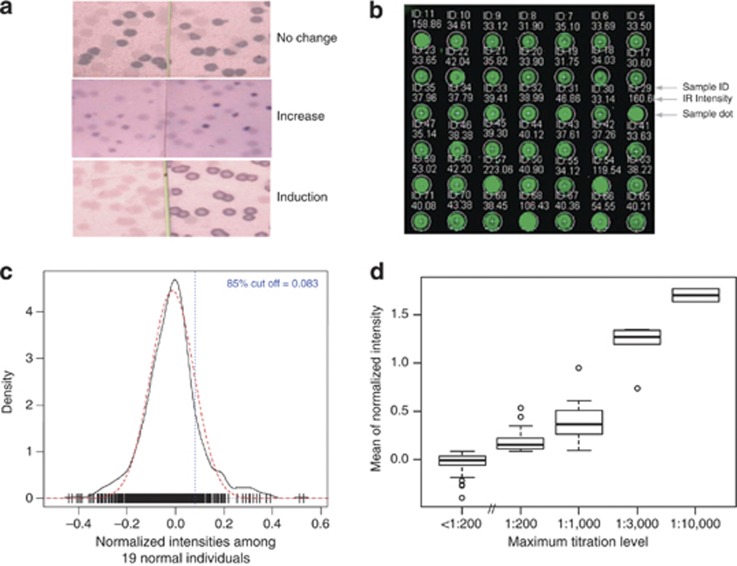
Development of a quantitative infrared immunoscreening assay, Quan-SEREX. (**a**) The presence of antibodies from patient serum samples against the candidate antigens were assessed in patient serum at pre-vaccine and week 12 post-vaccine time point using traditional SEREX technology. The response is scored as being ‘no change' (A, top) if there is no difference between the pre- and post-vaccine samples, ‘increased' (A, middle) if it is present in both pre- and post-vaccine samples, but the signal intensity is increased in the post sample, or ‘induced' (A, bottom) if the signal is only observed in the post-vaccine sample. (**b**) Quantitative dot blot assay: Each of the 14 antigens as well as controls (encoded in phage clone lysate) were doted in triplicates on a bacterial lawn and incubated with an IPTG soaked nitrocellulose membrane. This membrane is subsequently incubated with patient serum at 1:200. Phage binding to serum IgG was detected with infrared labeled anti-human IgG, and intensity of each spot corresponding to a particular phage clone was determined as described in Materials and Methods. (**c**) Threshold setting: Density of normalized intensities to candidate antigens among 19 normal donor individuals. The bell shape curve in black solid line presented the empirical density of normalized intensities, and the approximating normal distribution was shown in red dash line. Short vertical strips in the bottom indicate raw data of normalized intensities. Threshold was set as 85th percentile of approximating normal distribution and indicated in blue vertical dash line. (**d**) Normalized intensity determined by Quan-SEREX is a surrogate for antibody titer: Boxplot of normalized intensities at 1:200 titration by maximum dilution levels. The phage clone lysate were dotted on a set of four different nitrocellulose membranes that were later processed with four different dilutions (1:200, 1:3000, 1:10 000, and 1:30 000) of patient serum. Maximum dilution level was defined as the maximum titration where a phage was considered as recognized. <1:200 box indicated those phages that failed to be recognized in any titrations. Normalized intensity at 1:200 titration was explored with an increasing trend by maximum dilution levels (correlation coefficient=0.86 based on Spearman's approach).

Serological analysis of recombinant cDNA expression libraries of human tumors with autologous serum (SEREX) has been an exceptionally powerful technique for cancer antigen discovery.^20, 21, 22, 23^ We used a modified multi-step version of this technique to identify the specificity of induced anti-leukemia responses in patients with clinical responses to cancer vaccine therapy. Week 12 post-vaccination serum from five patients demonstrating clear clinical responses to initial K562/GVAX vaccination series (subjects 2, 4, 6, 9 and 18)^38^ was used to screen a K562 cDNA expression library as described elsewhere (Materials and Methods^23, 39^). After three rounds of sequential screening, purified monoclonal candidate clones were tested using pre- and week 12 post-vaccination serum to identify responses induced or augmented following vaccination (Figure 2a), and were seen in <20% of 19 normal donor sera tested. These clones had their cDNA inserts sequenced (Table 2) and were used in subsequent quantitative assessment. Two (RHAMM, DNAJA1) had been identified previously, using SEREX, as CML antigens^21, 22^ while we believe the remaining 12 represent novel LAAs.

### Quan-SEREX allows quantitative assessment of induced immunity

Direct infrared fluorescence imaging technology now allows quantitative detection assessment across a wide dynamic range with less background compared with conventional visible chemiluminescence approaches.^41^ Infrared image scanning (Materials and Methods, Figure 2b) was used to quantify the level of serum reactivity to the 14 antigens identified using traditional SEREX methodology. Sera from 19 normal healthy donors were first used to determine baseline performance characteristics of this technique. We approximated estimated background distribution of normalized intensities with a normal Gaussian density centered at the sample median, and with standard deviation an estimate of the standard deviation of 0.75 times the interquartile range of the normalized intensities of the 19 normal individuals (Figure 2c, red dashed line). Experimentally derived normalized intensities exhibited a similar bell-shaped curve (Figure 2c, black solid line) broadly consistent with the absence of leukemia antigen phage recognition in these unvaccinated healthy donors. A detection threshold at the 85th percentile (Figure 2c, vertical dotted line) of the approximated normalized intensity distribution (that is, 0.083) was selected to maximize sensitivity. Occasional deviation from expected distribution was observed above this threshold in normal healthy unvaccinated donors consistent with rare circulating autoantibodies. In this approach the threshold for antigen recognition was set based on the aggregate data of healthy donor responses to all antigens; based on our preliminary data exploration, this estimator is fairly robust to background noise, provides the advantage of computational benefits and appears to generate comparable results compared with setting individual thresholds for the recognition of each antigen. Good correlation (correlation coefficient=0.86) was observed between mean normalized intensity and traditional serum antibody dilution titer (Figure 2d).

### Decreased residual tumor burden following K562/GVAX immunotherapy is associated with the induction of high-titer IgG antibodies against multiple LAAs

**Figure 3:**
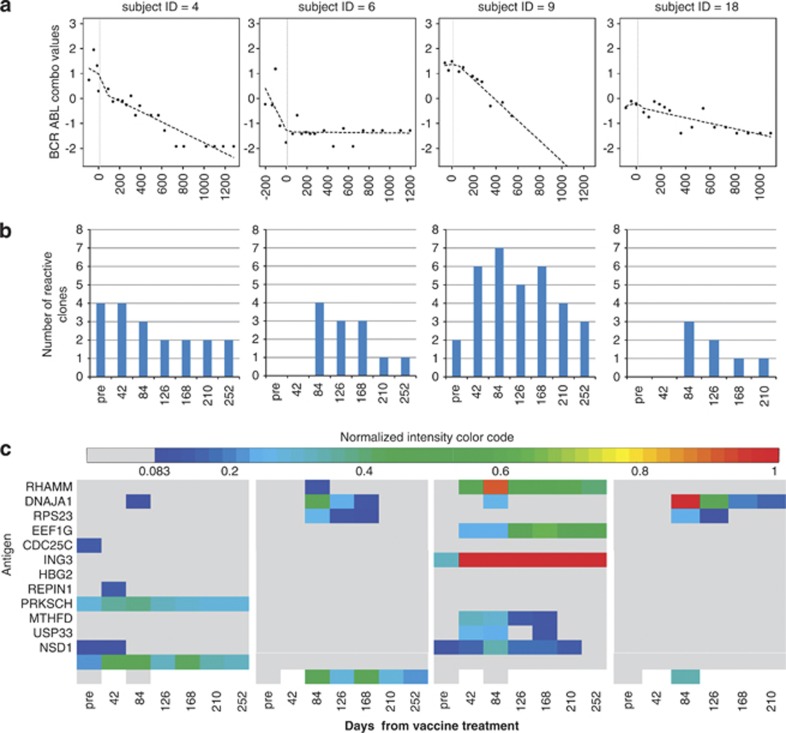
Clinical responders to K562/GVAX vaccination make novel high-titer IgG responses to a broad range of LAAs. (**a**) Residual CML disease burden as determined by BCR–ABL values as determined by RQ-PCR from patient PBMC mRNA before and after vaccination (vertical dashed line represents primary J0345 vaccination series). (**b**) Number of LAAs recognized by serum IgG in each patient before (‘pre') and after primary vaccination (time on *x* axis in days). (**c**) Quantitative measurement of LAA-specific serum IgG. Maximal ‘titer' of induced responses was observed between day 42 and 84 time points (that is, 6–12 weeks). Normalized intensity is color-coded (lower limit of detection, 0.083 in dark blue, maximal in red) according to the scale in Figure 4a.

**Figure 4:**
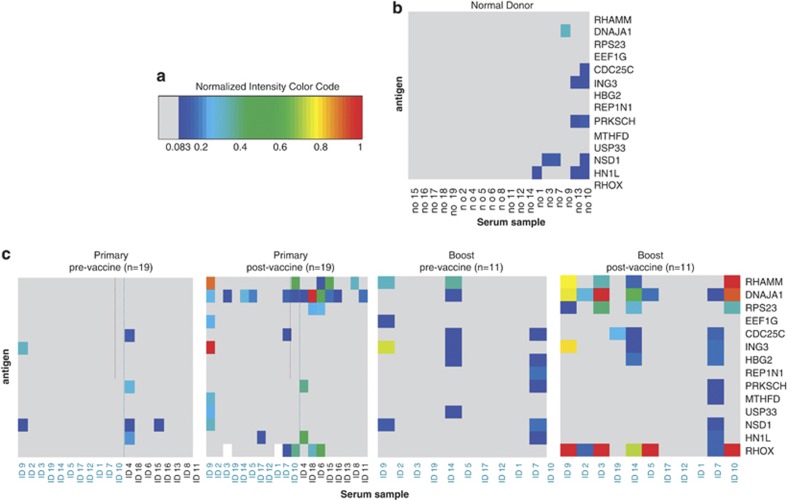
Discordant patterns of induced antibody response in identically vaccinated patients. (**a**) Color code for the heatmap of normalized intensity (∼titer) from dark blue (minimum/threshold of detection) to bright red (maximal, equal or greater to level seen for positive control). Antigens unrecognized at timepoint are coded in gray, missing samples are coded in white. (**b**) Normal donor reactivity against candidate LAAs. Threshold for detection was set at 85% of normal donor distribution to limit false positive results in patient dataset. As predicted by this, experimentally observed normal donor IgG reactivity to these 14 discovered LAAs was rare, and when responses were present they were low titer. (**c**) Normalized intensity heatmap of CML patient LAA-specific IgG responses before and after both primary and secondary vaccination. All patients received identical K562/GVAX whole-cell vaccine product at identical time points but exhibited markedly divergent responses.

Five patients were selected for an initial discovery cohort based on their unambiguously decreasing BCR/ABL mRNA transcript levels (Figure 3a) following initial K562/GVAX vaccination.^38^ Four of these five clinically responding patients were found to have induction of new leukemia-associated IgG responses following primary vaccination (Figure 3b), and two of these four were also shown to have augmentation of pre-vaccination leukemia antigen IgG antibody levels (Figure 3c). In total, only five unique antigens from our panel were recognized prior to K562/GVAX vaccination and responses to those same antigens were also seen in 5–15% of 19 healthy unvaccinated donors tested (Table 2). However, following vaccination, responses to one antigen (DNAJA1) were induced in all four patients, two patients (6, 18) developed new IgG antibodies to the same three antigens (DNAJA1, RPS23 and RHOX) and two patients (6, 9) made a novel response to the well-described leukemia antigen RHAMM.^22, 42, 43^ In total, post-vaccination responses were detected in 12 unique LAAs in the four patients (13 novel induced responses plus 3 augmented responses). The highest titer was noted at week 6–12 time points consistent with a response to vaccination. A greater diversity of antigens was observed to be recognized by IgG in early post vaccination (Figure 3b), although IgG titers against some antigens were elevated for over a year after vaccination (Figures 3c and 4).

### Highly individualized patterns of induced serological responses observed in identically vaccinated patients

This analysis was extended to the remaining 14 of 19 patients in the original (‘primary') vaccination trial and to the subsequent time-point samples in the subset of 11 patients who were offered an additional series of vaccinations (‘boost') due to a suboptimal clinical response to the original vaccination series (Figure 1). Following the initial vaccination series each of the remaining 14 individual patients made a response to between 0 and 3 antigens (median: 1). This compared to a median of 4 antigens (range 0–7) recognized in the discovery cohort consisting of those five patients making an unambiguous clinical response following vaccination (Figures 3 and 4).

**Figure 5:**
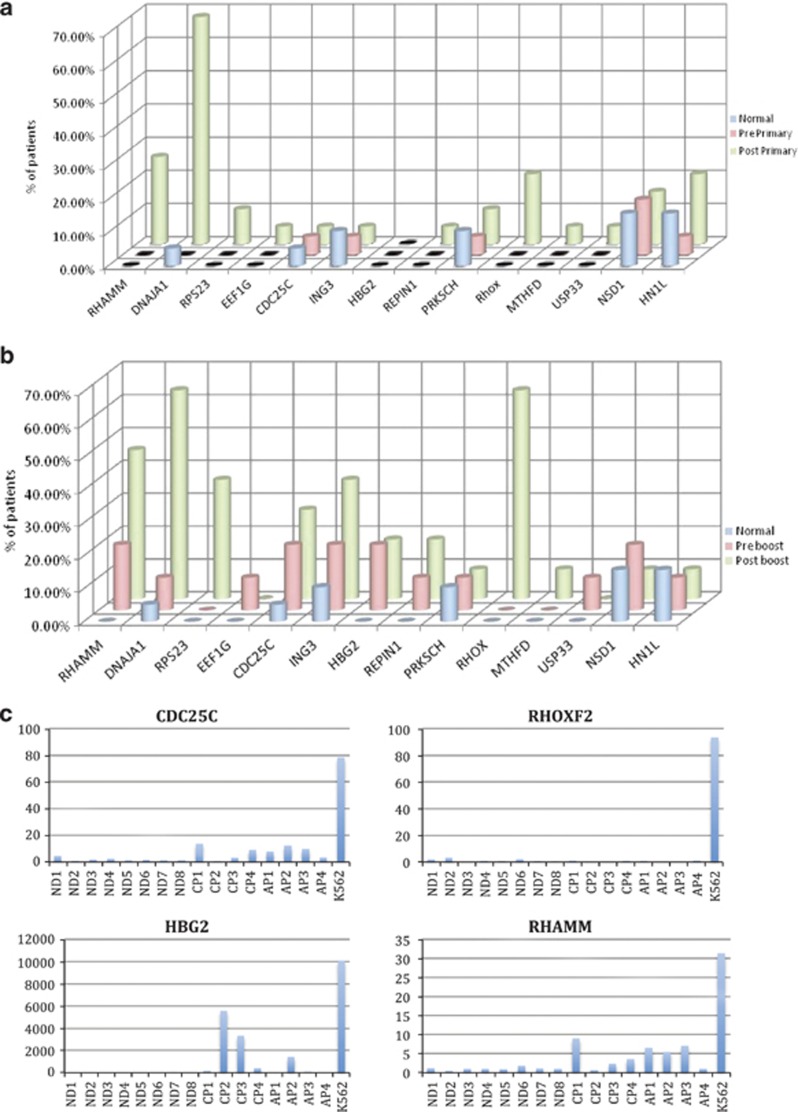
Relationship between antigen mRNA expression levels in CML and K562 to timing of development of IgG antibody response. Secondary ‘booster' vaccination could elicit IgG LAA responses in patients who did not make a response to that antigen after the initial vaccination series. Figure shows percentage of patients making LAA-specific IgG responses before and after either the first vaccination series (*n*=19 patients, (**a**) top panel) or booster vaccination series (*n*=11 patients, (**b**) middle panel). Number of normal donors (ND; *n*=19) making responses to the same antigen is shown for comparison in both panels. Antigens more commonly recognized by patients after a secondary ‘boost' vaccination series (CDC25C, RHOXF2, HBG2 and RHAMM) have higher levels of mRNA expression in K562 (shown as fold expression over the mean expression in 8 normal donor samples) compared with levels in peripheral blood levels from eight ND or eight patients with chronic (CP) or accelerated (AP) phase CML (**c**) lower panel).

**Figure 6:**
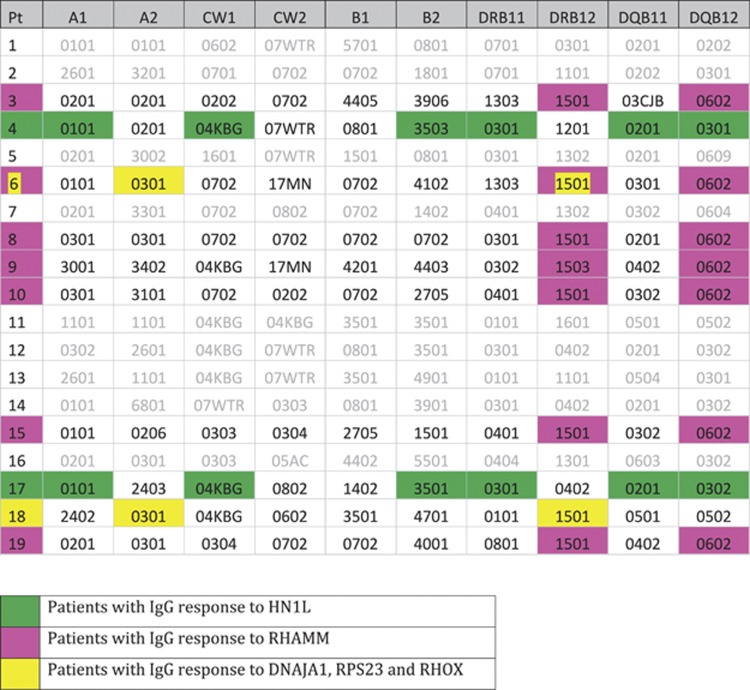
Role of patient HLA type in defining LAA IgG responses to K562/GVAX whole-cell vaccine immunotherapy. Molecular HLA typing was obtained from the Clinical Immunogenetics Laboratory of the Johns Hopkins University School of Medicine. The intra-patient variation in the induced IgG responses following vaccination with an identical K562/GVAX whole-cell vaccine product appears likely due not only to prior antigen experience and immunological memory but also to HLA differences between patients. Three examples are highlighted in the figure above; two clinical responders made responses to three of the same antigens (DNAJA1, RPS23 and RHOX). These patients shared both class I (HLA-A*0301) and class II alleles (DRB*1501) in their HLA haplotype. Additionally, 7 of the 19 patients in this study made a response to RHAMM. All had DRB15 and DQB*0602 alleles in their haplotype; indeed 7 of 8 patients with DRB15 and 7 of 7 patients with DQB*0602 in this study made responses to RHAMM. Finally, only two patients made an initial response to the antigen HN1L. They shared an almost identical HLA haplotype (HLA-A*0101, HLA-C*04KBG, HLA-B*035, HLA-DRB*0301 and HLA-DRQ*0201). No other patient in the study had this haplotype.

The induced IgG antibody response to vaccination in these 19 identically vaccinated patients was extremely heterogeneous (Figure 4). Two broad patterns of antigen-specific IgG induction were noted; in the first pattern, responses were seen in multiple patients even after a primary vaccine series and the number of patients responding did not change substantially after a secondary ‘boost' vaccination series (for example, DNAJA1). In the second pattern, the second ‘boost' vaccination did seem to increase the number of patients who made a new IgG response to an antigen (for example, RHOXF2B, HBG2 and CDC25C). We hypothesized that this was due to pre-existing immunological memory against the former class of antigens (from patients' own prior exposure to autologous CML) and that the expression of this latter class of ‘late' antigens would be higher in K562 cell line, from which the vaccine product was derived, than in primary CML-CP. We note that the mRNA expression of RHOXF2B (4/19 responses in primary vs 7/11 in boost), HBG (no responders at 12 weeks post-primary vaccination series, 2 responders at later time points) and CDC25C (1 responder post-primary, 3 responders post boost) all were overexpressed (5–40-fold) in K562 compared with primary CML-CP cells (Figure 5). Finally, surprisingly, despite the use of a whole-cell vaccination approach, some of the diversity observed in responses may be due to HLA restriction. Patients making IgG responses to the same antigen(s) after vaccination often shared multiple HLA alleles (Figure 6).

## Discussion

Although numerous LAAs have been reported,^2, 44, 45, 46^ less is known about the association between induced immunity to these antigens and successful clinical responses to immunotherapy.^23, 47^ Given the challenges in identifying the optimal LAAs needed to induce a clinical response, the difficulties in defining and validating individual peptide epitopes, the desire to not restrict immunotherapy availability to individuals with one particular HLA type and the significant time and resource costs of patient personalized autologous^48^ or matched allogeneic^49^ cellular products, we instead adopted a whole-cell vaccination strategy in an attempt to deliver a universal CML vaccine. We show here that CML patients with declining leukemia disease burden following vaccination with this K562/GVAX immunotherapy develop novel leukemia-specific high-titer immune responses not present in those patients prior to vaccination or in healthy donors.

There are several limitations to this study. First, we investigated only one part of the immune response—induced or augmented IgG antibody responses to LAAs; future work will concentrate on other antibody classes and T-cell responses. Second, this hypothesis-generating pilot study does not allow one to make statistical correlations between clinical responses, induced immune responses and/or patient characteristics. Third, patients on this study had only low levels of CML disease burden, which necessitated that our SEREX cDNA discovery library originate from the vaccine K562 cell line rather then autologous tumor and our gene expression studies use CML-CP obtained from third parties. Planned studies on myeloid leukemia antigen discovery in acute myeloid leukemia where the disease burden at study enrollment is higher will address both these limitations. Finally, consistent with prior SEREX work, a bacterial system for antigen expression was used, potentially resulting in false negative results due to prokaryotic glycosylation patterns. Future work (LQ, HIL) will attempt to develop a SEREX variant that utilizes a eukaryotic expression platform.

The high diverse nature of induced responses observed in this study may inform future vaccination strategies in cancer immunotherapy. Notably, no antigen was universally associated with IgG immune responses in all CML patients either at baseline, or following vaccination with an identical whole-cell vaccine. Following the initial vaccination series, 13 of 19 patients made responses to a single antigen (DNAJA1), but this was atypical with most antigens being recognized, on average, by 2 of 19 patients (11.35%).

In those 11 subjects who had a second series of booster vaccinations, two antigens (DNAJA1 and RHOX) elicited IgG immune responses in 7 of 11 patients, each antigen tested being recognized by 3 or 4 patients (31%) on an average. These antigens, together with RHAMM, accounted for the majority of new induced IgG responses seen after primary (22 of 28 new responses seen in 19 patients) and secondary (19 of 28 new responses seen in 11 patients) K562/GVAX vaccination series.

DNAJA1 (also known as HSJ 2, heat-shock 40-kd protein 4 or dnaJ homolog subfamily A member 1) was first identified in 1993^50^ and is now known to have an important and highly conserved role in the molecular chaperone system for protein folding, unfolding and degradation, acting as the primary co-chaperone of the constitutively expressed Hsp70 variant, Hsc70.^51^ Interestingly, overexpression of Hsp70 has been shown to be associated with resistance to imatinib in CML.^52^ Autoantibodies against HSPs and molecular chaperones have been described both in healthy and autoimmune disease states.^53^ Anti-DNAJA1 antibody responses have been found using SEREX screening of patient sera against autologous tumor cDNA libraries in patients with hepatocellular carcinoma,^54^ squamous cell carcinoma of the head and neck^55^ and in unpublished work deposited in the Cancer Immunome Database^56^ from lung, prostate and colon cancer patients. Responses to DNAJA1 (and other chaperone molecules) have also previously been described in CML patients by Greiner *et al.*^22^, using SEREX and a K562 cDNA library, where 3 of 10 CML patients tested but none of 19 untreated AML patients and none of 20 healthy volunteers had anti-DNAJA1 IgG responses. In comparison, in our study only a single healthy donor (of 19 tested) and no CML patients prior to vaccination (of 18 tested) had detectable IgG responses to DNAJA1; however, approximately two-thirds of patients developed anti-DNAJA1 IgG responses following K562/GVAX vaccination (Figure 5), including all four of the clinical responders having an induced antibody response after vaccination.

RHOXF2B (also known as RHOX homeobox family member 2B) is part of the family of the X-linked RHOX homeobox gene cluster that is an important epigenetically regulated component of transcriptional regulation of normal development and reproduction.^57^ RHOXF2B was found to be expressed in normal human testis tissue and a colon cancer cell line and could be induced by decitabine demethylation therapy in a human breast cancer cell line.^57^ In our study RHOXF2B was ∼100-fold overexpressed in the K562 cell line compared with normal donors or patients with chronic phase or accelerated phase CML (Figure 5). RHOXF2B was not recognized by IgG antibodies from the sera of normal donors or pre-vaccination CML patients but was recognized after primary (4 of 19 patients) and secondary vaccination series (Figure 4). RHOXF2B therefore appears to represent a novel, immunogenic and targetable, cancer testis antigen.

Finally, RHAMM (also known as HMMR, Hyaluronan-mediated motility receptor, CD168; IHABP) is a well-characterized CML-associated leukemia antigen.^21^ Previous work had shown serum specificity for this antigen in 5 of 16 CML patients tested.^21, 22^ In this study of 19 treated CML patients with minimal residual disease, none displayed IgG reactivity to RHAMM at baseline; however, a total of 7 patients responded after vaccination (5 after primary vaccination and another 2 after secondary vaccination).

Of those 9 antigens recognized only after vaccination (Table 1), only two are membrane-bound (RHAMM and DNAJA1), five are predominately cytoplasmic (HBG2, EEF1G, RPS23, USP33 and MTHFD2) and two are found in the nucleus (REPIN1 and RHOXF2B). In terms of function, four are involved in transcription regulation (RPS23, EEF1G and RHOXF2B) or control of replication (REPIN1) and two are cytosolic enzymes (MTHFD2 and USP33). No single antigen elicited induced or augmented IgG responses either in all patients or in the subset of patients having a clinical response following vaccination.

We have previously shown that whole-cell vaccination using a K562/GVAX product can lead to clinical responses in patients with residual CML after suboptimal responses to TKI therapy.^38^ In this study we demonstrate that such vaccination is associated with a highly diverse pattern of induced IgG responses to a broad and overlapping, but non-identical, spectrum of LAAs, 12 of which are described for the first time here. Clinical responders were able to generate high-titer responses to multiple antigens, and the induced responses were often long lasting. We additionally showed that a secondary K562/GM-CSF whole-cell vaccination series, given on average over 2 years after the primary vaccination series appears safe, well tolerated and was associated clinically with the lowest observed BCR/ABL transcript levels in 8 of 11 patients including two who became molecularly undetectable. This ‘booster' series could not only re-elicit and augment antigen-specific immune responses in previous responders but also induce antigen-specific IgG responses in those patients who did not respond to primary vaccination (Figure 4), particularly for those antigens with higher levels of expression in vaccine than primary tumor (Figure 5). Finally, patients making IgG responses to the same antigens often shared some or all of their HLA haplotype (Figure 6), a surprising observation in this whole-cell vaccine approach. We believe this to be the first description of this phenomenon in whole-cell vaccination and/or cancer vaccines. There is however precedent for induced antibody responses being favored by patients with certain distinct HLA alleles, with evidence from studies of vaccination against hepatitis B,^58^ rubella^59^ and HIV.^60^ If this observed relationship is determined to be statistically meaningful in subsequent work then future studies should aim to elucidate whether this represents a direct effect of altered MHC class II presentation and deficient T cells help in the induction of humoral immunity, an indirect effect through linkage disequilibrium with associated genes (such as the MHC class III region) or another mechanism that remains to be determined. In summary, these findings may have implications for future cancer vaccine immunotherapy approaches, particularly those attempting to target a single antigen or deliver a form of universal vaccine.

## Figures and Tables

**Table 1:** Patient response to ‘booster' K562/GM-CSF vaccination series in trial J0625

*ID*	*Age*	*Sex*	*Weeks between primary and boost vaccination*	*Time on TKI prior to boost vaccination series (wks)*	*BCR–ABL PCR pre-primary vaccination (J0345)*	*BCR–ABL PCR postprimary vaccination (J0345)*	*BCR–ABL PCR pre-boostvaccination (J0625)*	*BCR–ABL PCR post-boostvaccination (J0625)*	*Time from boost vaccination to lowest PCR (weeks)*
12	78	M	81	275	14	3.6	2	0.2	12
10	60	F	84	337	109	153	41	2.4	24
17	48	M	82	322	9.4	1.3	0.5	0.2	48
14	61	M	93	329	16	42	28	166	12
2	60	F	141	351	69	26	<0.05	0.3	36
1	63	M	122	266	28	<0.05	<0.05	<0.05	24
19	37	M	77	167	7.9	4.9	12	1	24
3	38	M	108	187	1.5	0.7	0.6	0.1	12
5	32	F	199	294	0.1	0	<0.05	0	48
7	68	F	107	305	0.3	0.2	0.07	0.1	36
9	49	M	131	233	69	6.4	0.08	0	48

Patient demographic and clinical response data for those patients enrolled on both initial (J0345) and boost (J0625) clinical protocols. All patients remained on tyrosine kinase inhibitor (TKI) therapy during and between both protocols. PCR was performed every 6 weeks during each study using patient peripheral blood as previously described^38^ and is reported in terms of number of BCR–ABL fusion transcripts detected per 1000 ABL reference transcripts. Orange (<0.05) represents non-quantifiable levels of minimal residual disease, red (0) denotes no evidence of BCR–ABL fusion transcript detected.

**Table 2:** Identification of leukemia-associated antigens

*Antigen*	*Other name*	*Patient responses pre-vaccine (*N*=18)*	*Patient responses post vaccine (*N*=19)*	*Seen in healthy donors (*N*=19)*
RHAMM	Hyaluronan-mediated motility receptor	0	5	0
DNAJA1	DnaJ (Hsp40) homolog, subfamily A, member 1	0	13	1
RPS23	Ribosomal protein S23 (RPS23)	0	2	0
EEF1G	Eukaryotic translation elongation factor 1 gamma	0	1	0
REPIN1	Replication initiator 1, transcript variant 4	0	1	0
RHOX	Rhox homeobox family, member 2B (RHOXF2B)	0	4	0
MTHFD	Methylenetetrahydrofolate dehydrogenase (NADP+ dependent) 2	0	1	0
USP33	Ubiquitin specific peptidase 33 (USP33), transcript variant 1	0	1	0
HBG2	Hemoglobin, gamma G ( HBG2)	0	2*	0
				
*Antigens to which antibodies were present at baseline pre-vaccination*				
CDC25C	Cell division cycle 25 homolog C	1	1	1
ING3	Inhibitor of growth family, member 3, transcript variant 1	1	1	2
PRKCSH	Protein kinase C substrate 80K-H	1	2	2
NSD1	Nuclear receptor binding SET domain protein 1, transcript variant 1	3	3	3
HN1L	Hematological and neurological expressed 1-like	1	4	3

SEREX (serological analysis of recombinant cDNA expression libraries of human tumors with autologous serum) was used to identify the specificity of induced anti-leukemia responses in patients with clinical responses to cancer vaccine therapy. Serum from five clinical responders was screened against a K562 expression library, followed by three rounds of subsequent screening to isolate a monoclonal phage for sequencing. Purified targets from the discovery cohort were sequenced and also used to screen sera from both normal donors (*n*=19) and pre (*n*=18, one sample missing)- and post (*n*=19)-primary vaccination patients. Two patterns of reactivity were observed; antigens to which IgG responses were seen in vaccinated patients but were not seen in either normal donors or patients prior to vaccination (9 of 14 antigens identified), and second, those antigens to which IgG responses were seen in all three groups (5 of 14 antigens identified). *HBG specific IgG responses were observed late in ‘pre-boost samples' only.

## Notes

HIL is an employee of Roche and a part-time faculty member at Johns Hopkins University. All other authors declare no conflict of interest.
